# Prospective observational cohort study of ‘treatment as usual’ over four years for patients with schizophrenia in a national forensic hospital

**DOI:** 10.1186/s12888-018-1862-0

**Published:** 2018-09-08

**Authors:** Melanie S. Richter, Ken O’Reilly, Danny O’Sullivan, Padraic O’Flynn, Aiden Corvin, Gary Donohoe, Ciaran Coyle, Mary Davoren, Caroline Higgins, Orla Byrne, Tina Nutley, Andrea Nulty, Kapil Sharma, Paul O’Connell, Harry G. Kennedy

**Affiliations:** 1 National Forensic Mental Health Service, Central Mental Hospital, Dundrum, Dublin, Ireland; 2 Department of Psychology, Maastricht University, Maastricht, The Netherlands; 3 Department of Psychiatry, Trinity College, Dublin, Ireland; 4 Department of Psychology, National University of Ireland Galway, Galway, Ireland

**Keywords:** Forensic psychiatry, Treatment, Cognition, Violence risk, DUNDRUM toolkit

## Abstract

**Background:**

We evaluated change in response to multi-modal psychosocial ‘treatment as usual’ programs offered within a forensic hospital.

**Methods:**

Sixty nine patients with a diagnosis of schizophrenia or schizoaffective disorder were followed for up to four years. Patient progress was evaluated using the DUNDRUM-3, a measure of patient ability to participate and benefit from multi-modal psychosocial programs and the HCR-20 dynamic items, a measure of violence proneness. We report reliable change index (RCI) and reliable and clinically meaningful change (RMC). We assessed patients’ cognition using the MCCB, psychopathology using the PANSS. The effect of cognition and psychopathology on change in DUNDRUM-3 was examined using hierarchical multiple regression with age, gender, and baseline DUNDRUM-3 scores.

**Results:**

The DUNDRUM-3 changed significantly (*p* < 0.004, d = 0.367, RCI 32% of 69 cases, RMC 23%) and HCR-20-C (*p* < 0.003, d = 0.377, RCI 10%). Both cognition and psychopathology accounted for significant variance in DUNDRUM-3 at follow up. Those hospitalized for less than five years at baseline changed more than longer stay patients. Mediation analysis demonstrated that the relationship between cognition and change in violence proneness (HCR-20-C) was both directly affected and indirectly mediated by change in DUNDRUM-3.

**Conclusions:**

Change in response to multi-modal psychosocial programs (DUNDRUM-3) reduced a measure of violence proneness over four years. Forensic in-patients’ ability to benefit from psychosocial treatment appears to be a function of the outcome measure used, unit of measurement employed, degree of cognitive impairment, psychopathology, and length of stay. Lower risk of re-offending may be partially attributable to participation and engagement in psychosocial interventions.

## Background

Forensic Mental Health Services (FMHS) provide treatment and care for the minority of people with mental disorders who come in contact with criminal justice services or require specialized care [1, 2]. Although there is evidence that forensic patients have a lower risk of re-offending compared to prisoners, it is not known whether the lower risk arises from clinical interventions [3].

The effectiveness of pharmacotherapy is well documented for treating symptoms [4]. There is less evidence for improved real world function though there is evidence that pharmacotherapy can reduce violence [5, 6]. However many community and forensic patients do not adhere to their medication when discharged. Psychosocial interventions may improve not only adherence to medication [5–8] but also a range of other ‘real world’ functional outcomes. Currently there is mixed evidence that forensic patients benefit from psychosocial interventions for non-adherence, refractory symptoms and violence risk [9–13]. Randomized controlled trials in this field are difficult and there are few available as guides [12, 13]. Paradoxically pharmacotherapy may compromise the effectiveness of psychosocial interventions to some extent by impairing neurocognition and functioning [14, 15].

In a review of effective interventions for reducing violence and aggression, McGuire [16] recommended that because of the complexity of the problem, it appears advisable to research multimodal interventions only, with greater intensity of treatment and improved targeting. Similarly, Wampold [17] reviewed the common factors in psychotherapy and concluded that common factors such as alliance, empathy, expectations, cultural adaptation and therapist differences have large effect sizes in meta analyses, while specific factors such as treatment differences, adherence and competence have smaller effects.

Because measurement of response to psychosocial programmes is difficult, we have designed and validated a measure of multi-modal treatment, the DUNDRUM-3 [18]. Accordingly the form and content of the DUNDRUM-3 programme completion scale [18] corresponds to a framework for assessing response to multi-modal treatments relevant to reducing violence broadly in accordance with McGuire [16]. The DUNDRUM-3 is rated according to progress in cycle of change [19], therapeutic engagement, recovery [20], Maslow’s hierarchy [21] and cultural engagement, a synthesis of theories of therapeutic effectiveness and change, broadly based on Wampold [17]. The scoring system for the DUNDRUM-3 programme completion scale [18] is designed to assess readiness to move from more secure to less secure locations [22–24] and is therefore rated in clinically meaningful units, relevant to outcomes.

Factors to be considered when evaluating psychosocial treatments include the method of measurement, the duration of treatment required, and cognitive impairment. Many forensic services evaluate progress using violence risk assessments such as the Historical-Clinical Risk management-20 (HCR-20) [25]. Meta-analyses demonstrate the validity of the HCR-20 for predicting violence [26, 27]. However, the clinical and risk items of the HCR-20 may not be sensitive to change [28–31]. The HoNOS and HoNOS-SECURE also appear insensitive to change [31, 32].

There are also questions concerning the duration and intensity of interventions to optimize outcomes [14–16]. Forensic patients may require longer durations of treatment than community patients. Many patients are hospitalized within forensic services for more than five years [33–38]. Reasons for this are complex, with legal reasons balanced by clinical complexity [31, 36, 38] and treatment needs [22, 23].

Cognitive impairment is likely to be an important determinant of ability to benefit from interventions in patients with schizophrenia [9, 39, 40]. Cognitive impairments are associated with many mental disorders including schizophrenia, autism, dementias, bipolar disorder and depression [41–43]. Patients with cognitive impairments may struggle to focus on relevant information and to process, store, and utilize the information when required [44]. Forensic patients who have been hospitalized for longer periods may be refractory to pharmacological and psychosocial treatments as a consequence of cognitive impairment [23, 45, 46]. Because many studies find that a mean or median length of stay in medium security is approximately five years [33–38] and because this was close to the median length of stay in this sample, we took five years as a likely distinction between those who were responsive to treatment relevant to length of stay in a forensic hospital and those who were less responsive to relevant treatment.

Because forensic services are costly and because of the limitations of pharmacological treatments, establishing the effectiveness of psychosocial treatments within this setting is a priority [47]. We hypothesized that:

1. forensic patients can benefit from a range of psychosocial treatment programs offered at a forensic hospital.
2. patients hospitalized for less than five years benefit more from treatment than patients who were hospitalized for more than five years.
3. cognitive impairment accounts for a significant amount of the variance in patients’ ability to benefit from treatment programs.
4. the DUNDRUM-3 will be sensitive to changes that are relevant to violence proneness.

## Methods

### Setting and sample

This was part of a naturalistic four-year observational cohort study beginning in December 2012 ending in December 2016 carried out at the Central Mental Hospital (CMH), the only medium and high secure forensic hospital in the Republic of Ireland [48]. At the time of the study, there were 94 secure beds on campus and 13 beds in the community for those on leave or conditionally discharged. These were stratified according to therapeutic security and risk-need principles, with high staff-to-patient ratios of multi-disciplinary care including nursing, psychology, social work, occupational therapy and psychiatry [3, 48–51].

**Fig. 1:**
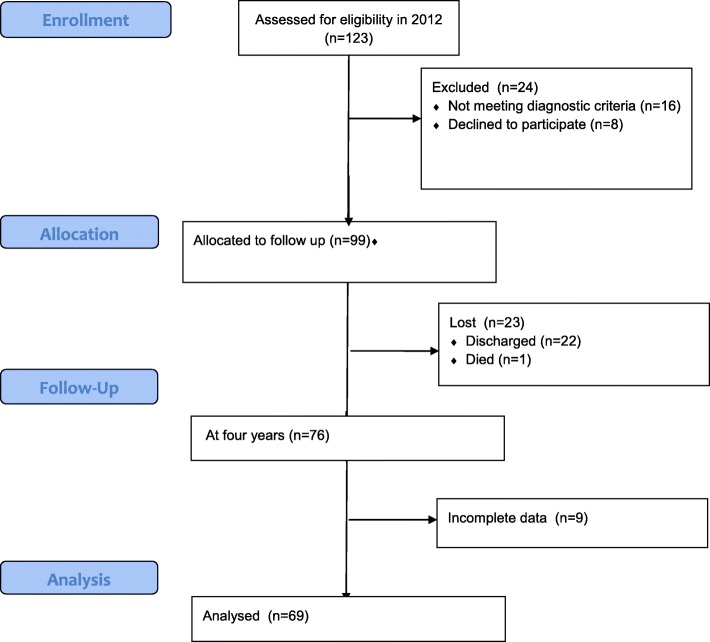
Consort flow diagram

Only patients with a DSM-IV diagnosis of schizophrenia or schizoaffective disorder and who were in-patients at the start of the study were included (SCID) [52]. Figure 1 (CONSORT flow diagram) describes the sample and attrition at follow-up. In total 69 patients participated and completed all measures, 58 with schizophrenia and 11 with schizoaffective disorder.

Most patients (82.6%) stayed in the study until the end of 2016. For those who did not stay until the end of 2016 the last assessment was taken as follow-up measure. Demographics are summarized in Table 1. The mean DUNDRUM-1 triage security score was 3.03 (SD 0.47) indicating that all had a history of serious violence and the group had a need for treatment in conditions of medium or high therapeutic security at the time of admission.

**Table 1:** Demographics and sample characteristics

	Full sample (*n* = 69)			Less than 2112 days (*n* = 35)			More than 2112 days (*n* = 34)		
	$\overline{x}$	M	S.D.	$\overline{x}$	M	S.D.	$\overline{x}$	M	S.D.
Age	39.72	38.00	11.13	34.11	33.00	6.91	45.50	43.50	11.77
DUNDRUM-1 triage security (0–4)	3.03	3.00	0.47	2.95	3.00	0.55	3.07	3.06	0.38
Length of stay at baseline (years)	7.94	5.00	8.71	2.54	3.00	1.96	13.50	11.00	9.46
Length of follow-up (years)	3.42	4.00	1.32	3.03	4.00	1.62	3.82	4.00	0.76
MCCB composite (t-score)	21.09	21.00	14.21	19.68	16.00	14.34	22.53	23.50	14.14
PANSS total score	64.27	60.00	21.80	67.74	63.00	25.79	60.71	58.50	16.36
PANSS positive	14.31	12.00	7.98	15.41	13.00	9.38	13.22	11.50	6.23
PANSS negative	19.08	18.50	7.86	18.69	18.00	8.25	19.47	19.00	7.56
CPZeq (mg/day)	529.57	467.00	341.92	526.66	500.00	284.27	532.58	458.50	397.03

*Note*: $\overline{x}$ mean, *SD* standard deviation, *M* median, *MCCB* MATRICS consensus cognitive battery, *PANSS* Positive and Negative Syndrome Scale, *CPZeq* Chlorpromazine equivalent

During the period of this naturalistic observational study, patients participated in a range of treatments routinely offered within forensic mental health services that could be described as ‘treatment as usual’. The treatments target seven pillars of care which include programs focused on 1) physical health, 2) mental health, 3) drugs and alcohol, 4) problem behaviors, 5) self-care and activities of daily living, 6) education occupation and creativity, 7) family and social networks. Patients progress from less demanding to more demanding programs as operationalized within the DUNDRUM Toolkit manual [18]. We believe these can be regarded as in keeping with multi-modal treatment [16].

### Measures

#### Cognitive functioning

Cognition was assessed using the composite score of the MATRICS Consensus Cognitive Battery (MCCB) [53] for patients with schizophrenia [54]. The MCCB covers seven cognitive domains affected by schizophrenia: processing speed; attention /vigilance; working memory; verbal learning; visual learning; reasoning and problem solving; social cognition. In validation studies and in antipsychotic trials of stable patients, the MCCB demonstrated excellent reliability, minimal practice effects and significant correlations with measures of functional capacity [54]. The MCCB was administered by masters level assistant psychologists trained in its use who worked independently of patients’ multidisciplinary teams (MDT). The MDTs were blind to the MCCB results. Results are reported as t-scores (normal population mean = 50, standard deviation = 10).

#### Presence of positive and negative symptoms

The total score of the Positive and Negative Syndrome Scale (PANSS) [55] was used for the assessment of the severity of symptoms of schizophrenia. The PANSS was assessed by masters level assistant psychologists trained in its use. The inter-rater reliability of the PANSS is well established in this setting [50, 51]. Those rating the PANSS were blind to the MCCB and DUNDRUM-3 ratings for the same patients.

#### Risk assessment

The Historical Clinical Risk Management Scale-20 (HCR-20; Version 2) [25] is a structured professional judgment tool for assessing risk of violence, or violence proneness. The HCR-20 is among the most widely used violence risk assessment schemes [26, 27]. The HCR-20 contains ten historical or static items, five current or clinical items (HCR-20-C) and five future risk items (HCR-20-R). Both the clinical and risk items are thought to be dynamic in nature in that they can change over time and are amenable to therapeutic intervention and evaluating outcome. Because the historical items are static in nature only the dynamic scale (HCR-20-D) consisting of the sum of the HCR-20-C and HCR-20-R and the clinical and risk scales themselves will be used as outcome measurements. Each item is scored 0 to 2 (absent, possible and present) so that the sub-scales HCR-20-C and HCR-20-R each has a range 0 to 10 and the HCR-20-dynamic has range 0 to 20. In decision making, these are used as the basis for a structured clinical judgment; they are not interpretable as units of meaningful change. The inter-rater reliability and validity of clinically rated HCR-20 V2 is well established in this setting [50, 51]. Those rating the HCR-20 were blind to the ratings of MCCB and PANSS for the same patients and blind to the DUNDRUM-3 ratings of the consultant psychiatrist.

#### Program completion

The DUNDRUM-3 programme completion scale measures participation, engagement and change in relation to seven treatment domains [18]. It is an outcome measure, and is therefore compatible with any set of hospital programmes. This means that the DUNDRUM-3 can be used in any forensic hospital or other mental health settings and allows comparison across services. Progress is regularly reviewed at MDT case conferences. The DUNDRUM-3 allows for patients to be positively or negatively scored according to sustained evidence of having benefited from each treatment domain. This has good inter-rater reliability and internal consistency [56] and has been validated against ‘real world’ outcomes including moves to less secure places [22], conditional discharge [23] and in-patient violence [51].

##### Treatment as usual

The DUNDRUM-3 programme completion scale deliberately does not require any specific manualized or other treatment programmes, since individual patients will have varying needs, and there is no current basis to prefer any one manualised programme over another. Nor is there evidence that manualised programmes are necessarily better than less structured programmes [17], when alternative structures may be more effective, including errorless learning and scaffolding [57]. Each of the seven items of the DUNDRUM-3 programme completion scale lists representative programmes where useful [18, 31, 56]. During the period studied, pillar I ‘physical health’ was managed in accordance with national standards [58, 59] while other relevant standards have also been summarized [60]. This included a six-monthly full review by family practitioner and primary care nurse and access to a metabolic physician along with dentist, dietician, physiotherapy and other allied health professionals; pillar II ‘mental health’ programmes are based on internationally recognized treatment [61] and medication protocols [62] up-dated as appropriate in the light of evidence based guidance, the Wellness Recovery Action Plan [63] and a range of modern evidence-based psychotherapies including cognitive remediation [57], metacognitive therapy [64] and cognitive behaviour therapies; pillar III the ‘substance misuse recovery’ programme included a four session brief information group then an eight session education group [65, 66], a full 28 session relapse prevention programme [67] and an aftercare self-help group; pillar IV ‘problem behaviours’ are delivered in the form of a self-risk management group programme which includes a 33 session first phase in six modules [68] and a second phase framework for behavioural analysis which typically includes an analysis of the most serious index offence in one to one work [69]; pillar V ‘self-care and activities of daily living’ are delivered in accordance with principles of the Model of Human Occupation (MOHO) [70]; pillar VI ‘education’ was delivered in accordance with national curricula while ‘occupation and creativity’ were delivered according to MOHO and educational principles; pillar VII ‘family and intimate relationship’ therapies were delivered variously by mental health social workers and trained family therapists.

All programmes were organised into three phases: introductory phases consisting of short course interventions often oriented around giving information suitable for delivery during acute phases of illness, substantive phases delivered after the acute phases of illness and self-maintenance phases for follow-up. All were assessed for successful engagement according to five over-arching principles including cycle of change [19], engagement, recovery [20], hierarchy of needs [21] and cultural integration (Table 2) [18]. Frequency and intensity of sessions has been described above. We regularly audited hours of engagement in structured therapeutic activities in accordance with the quality standard of 25 h per week [71] and found that this was consistently met for 85% of patients. However during the period of this study we were not able to audit hours in each domain for each patient.

**Table 2:** Theoretical background for scoring DUNDRUM-3

Units of meaningful change	Cycle of change [19]	Engagement [18]	Recovery [20]	Hierarchy of needs [21]	Spiritual and cultural integration
4: not ready to move down a level of security	Pre-contemplation.	Reluctance/ resistance	Moratorium	Physiological needs	Alienated
3: ready for a move e.g. from high to medium security	Contemplation & preparation, ambivalence	Passive engagement	Awareness, finding hope	Safety and basics of life	Can engage only in ‘trading’ interactions on an impersonal or binary basis.
2: ready for a move e.g. from medium to low security	Action / decisional balance	Active engagement	Preparation, search for personal resources and external help, taking responsibility	Friendship and family relationships	Accepts and commits to communal and social customs and affiliations.
1: ready for a move to supported community living e.g. conditional discharge or community treatment order	Maintenance, supported	Positive engagement	Rebuilding, taking positive steps, establishing a positive identity	Self-esteem, confidence, social standing	Accepts and commits to communal and social concepts of value and virtue.
0: ready for independence	Maintenance, stability	Taking personal responsibility	Growth / sense of control / looking forward, finding meaning and purpose in life	Self-actualisation Expresses self in creative and communicative ways.	Self-transcendence Fulfils roles and shares own resources (time, effort) for communal good

##### Clinically meaningful units of change

DUNDRUM-3 programme completion scale is rated as seven items corresponding to the seven pillars of care and treatment listed above. Each item has five points (0–4), calibrated in clinically meaningful units of change. DUNDRUM-3 scores which are mostly 4 indicate that a move to a less secure place is not yet appropriate, 3 indicates a move from high to medium security, 2 indicates a move from medium to low security, 1 indicates readiness for discharge to community settings and 0 corresponds to independence [18, 56]. When all seven items are summated and dividing by seven this returns a mean item score that has the same range as the subscales (0 to 4) [56] again corresponding to units of clinically meaningful change. The DUNDRUM-toolkit was found to meet most criteria for routine outcome measures in FMHS [24]. The use of the mean item score has been documented elsewhere [18, 22, 23, 51, 56, 72, 73]. The DUNDRUM-3 was completed by treating consultant psychiatrists all of whom act as trainers in its use. Those rating the DUNDRUM-3 were blind to the MCCB and PANSS ratings and blind to the current HCR-20 ratings.

### Statistical analysis

Data were analyzed using SPSS-24. All measures were screened for outliers and normality. In total, four cases of the HCR-20-D were labeled as outliers identified by the outlier labeling rule and were winsorized [74, 75].

Paired sample *t*-tests were used to investigate whether HCR-20 dynamic scales or mean DUNDRUM-3 scores changed significantly from baseline to follow-up and within-group effect sizes (Cohen’s *d*) were calculated where > 0.2 is small, > 0.4 is moderate and > 0.8 is large [75]. Additionally, a Reliable Change Index (RCI) [30, 32, 76] was calculated for the DUNDRUM-3 and the HCR-20 dynamic scales to determine whether the magnitude of change was statistically reliable and not the result of measurement error. The RCI is a standard means of determining if a measured change is beyond that expected by measurement error. Because the DUNDRUM-3 is calibrated in clinically meaningful units of change, when the RCI is less than one such clinically meaningful unit, then a change of one unit in the mean item DUNDRUM-3 score is a reliable and meaningful change (RMC). The number and percentages of patients achieving RCI and RMC is also reported.

The median length of stay at baseline was 2122 days, in keeping with the international average of approximately five years [31, 33–37]. To explore the effect that length of stay at baseline had on change, a median split was applied. Paired samples t-tests were again applied to sub-groups with above or below median length of stay for the HCR-20 dynamic scales and the mean DUNDRUM-3 scores.

Hierarchical multiple regression was performed to explore factors influencing change in the mean DUNDRUM-3. MCCB and PANSS were entered in two steps while controlling for age, gender and mean DUNDRUM-3 baseline score. PANSS was entered in the final step for two reasons: first, neurocognitive decline occurs prior to onset of psychosis and is relatively static whereas psychopathology is more amenable to treatment [39], and second because the PANSS is contaminated by function. To investigate predictors in the group of patients who stayed in hospital for less than 2112 days at baseline, the regression was repeated for this subgroup.

**Fig. 2:**
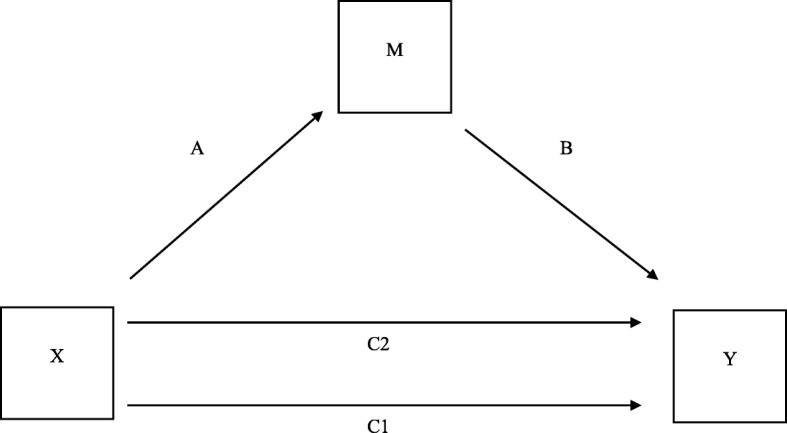
This figure is labelled to explain the meaning of the columns in Table 5. C1, Direct effect of X on Y, before mediation via M; C2, direct effect of X on Y after mediation via M; A, indirect effect of X on Y mediated via M; B, direct effect of M on Y, adjusted for X

The mediation effects between MCCB, change in mean DUNDRUM-3, and change in HCR-20-C were explored using Hayes process macro model 4 in SPSS [77] with 10,000 bootstrapped samples (Fig. 2).

HCR-20-C baseline scores and mean DUNDRUM-3 baseline scores were added as covariates.

## Results

### Hypothesis 1: Change in the mean DUNDRUM-3 and the HCR-20 dynamic scales

Mean DUNDRUM-3 scores for the total sample reduced by a mean of 0.29 points (range, 0 to 4) over the four-year period. This change was statistically significant (*t*(68) = 2.98, *p* = 0.004, d = 0.367). There was no significant reduction in the HCR-20 dynamic scale (*t*(68) =1.555, *p* = 0.125). The HCR-20 dynamic scales were further investigated by applying paired sample t-tests to the HCR-20-C and HCR-20-R scales separately. HCR-20-C reduced by an average of 0.81 points (range, 9) over the four year period, which was statistically significant (*t*(68) = 3.125, *p* = 0.003, d = 0.377). There was no significant reduction of the HCR-20-R (*t*(68) = − 0.436, *p* = 0.664) (Table 3).

**Table 3:** Baseline, Follow-up and change over 4-year period

	Baseline			Follow-up			difference					*RCI*		*RMC*	
	$\overline{x}$	SD	M	$\overline{x}$	SD	M	$\overline{x}$	SD	M	*p*	*d*	n	%	n	%
Full sample *n* = 69															
D-3	2.35	0.95	2.42	2.07	1.13	1.86	0.29	0.79	0.14	0.004	0.367	22	31.8	17	23
HCR-D	7.98	4.48	8.00	7.29	4.57	6.00	0.70	3.71	0.00	0.125	0.187	7	10.1		
HCR-C	4.52	2.77	5.00	3.71	2.62	3.00	0.81	2.16	0.00	0.003	0.377	7	10.1		
HCR-R	3.46	2.10	3.00	3.56	2.42	3.00	−0.10	1.93	0.00	0.664	−0.053	3	4.3		
Less than 2112 days (*n* = 35)															
D-3	2.62	0.96	3.00	2.29	1.16	2.14	0.33	0.87	0.14	0.030	0.392	13	37.1	11	31.4
HCR-D	8.57	5.13	9.00	7.57	4.81	7.00	1	4.07	1.00	0.156	0.246	4	11.4		
HCR-C	4.94	3.17	5.00	3.91	2.41	3.00	1.03	2.24	1.00	0.010	0.495	5	14.3		
HCR-R	3.63	2.28	3.00	3.66	2.76	3.00	−0.28	2.05	0.00	0.935	−0.127	2	5.7		
More than 2112 days (*n* = 34)															
D-3	2.08	0.88	2.07	1.84	1.07	1.71	0.24	0.72	0.14	0.065	0.339	9	26.4	6	17.1
HCR-D	7.38	3.68	7.00	7.00	4.37	6.00	0.38	3.34	0.00	0.509	0.116	3	8.8		
HCR-C	4.09	2.26	3.00	3.47	2.46	3.00	0.59	2.08	0.00	0.108	0.302	2	5.9		
HCR-R	3.29	1.91	4.00	3.50	2.49	3.00	−0.18	1.83	0.00	0.578	−0.119	1	2.9		

*Note*: $\overline{x}$ mean, *SD* standard deviation, *M* median, *D-3* DUNDRUM-3 Programme Completion scale, *HCR-D* HCR-20 dynamic items, *HCR-C* HCR-20 clinical items, *HCR-R* HCR-20 risk item, *RCI* Reliable Change Index, *RMC* Reliable and Meaningful Change

The RCI for the mean DUNDRUM-3 was 0.81. It follows that the clinically meaningful change in mean DUNDRUM-3 score of one whole unit is always more than the reliable change index and is generally also a clinically meaningful change (RMC) representing a step from one level of therapeutic security to the next. RCI for the HCR-20-C was 3.35, a score that cannot be related to clinically meaningful change in the same way. For the mean DUNDRUM-3, 22 patients (30.4%) had a RCI change of 0.81 or more and 17 (23%) had a reliable and clinically meaningful change (RMC) of 1 or more. Seven patients (10.1%) showed RCI change when measured by the HCR-20-C.

### Hypothesis 2: Change in mean DUNDRUM-3 and HCR-20-C in shorter and longer stay subgroups

Comparing baseline assessment and follow-up assessment of mean DUNDRUM-3 scores, paired t-tests revealed that there was a significant change in those who were in the hospital for less than 2112 days at baseline (*n* = 35; *t*(34) = 2.270, *p* = 0.030, d = 0.392) but not for the group of patients who were in the hospital for more than 2112 days at baseline (*n* = 34; *t*(33) = 1.908, *p* = 0.065). The HCR-20-C also changed significantly for the sub-group with shorter length of stay at baseline (*p* = 0.01, d = 0.495) but not for the longer stay sub-group. The two subgroups did not differ significantly in MCCB score, gender or baseline DUNDRUM-3. Patients who stayed in the CMH for more than 2112 days were significantly older and scored lower on the PANSS total score.

### Hypothesis 3: Cognitive impairment and symptom severity as predictors of change in mean DUNDRUM-3 over 4-year period in total sample

In hierarchical multiple regression the total model accounted for 19.2% (*F*(5, 53) = 4.235, *p* = 0.002) of the total variance in mean DUNDRUM-3 change scores. After controlling for age, gender, and mean DUNDRUM-3 baseline score, cognition explained 9.9% of the total variance of change in mean DUNDRUM-3 (*F-*change (1, 64) = 7.689, *p* = 0.007). Psychopathology added 7.4% to the total variance explained in mean DUNDRUM-3 change scores (*F-*change (1, 63) = 6.207, *p* = 0.015) (Table 4).

**Table 4:** Summary of hierarchical linear regression model of the effect of predictors on change on program completion mean (D-3) at 4-year follow-up

	Model summary for each step	
	Adjusted *R*^2^	*R*^2^ change
Full sample (*n* = 69)		
Age, gender, mean D-3 baseline	0.037	0.079
+ MCCB	0.126	0.099**
+ PANSS	0.192	0.074*
Less than 2112 days (*n* = 35)		
Age, gender, mean D-3 baseline	−0.010	0.079
+ MCCB	0.193	0.209**
+ PANSS	0.304	0.118*

Note: *p ≤ 0.05, **p ≤ 0.01. *MCCB* MATRICS Consensus Cognitive Battery, *PANSS* Positive and Negative Syndrome Scale, *D-3* mean DUNDRUM-3 Program Completion scale

In the final model, three measures were statistically significant predictors of change in mean DUNDRUM-3 score, with mean DUNDRUM-3 baseline score having the highest beta value (β = 0.523, *p* = 0.001), followed by psychopathology (β = − 0.351, *p* = 0.015), and cognition (β = 0.338, *p* = 0.015).

### Hypotheses 3 and 2: Predictors of change in DUNDRUM-3 over 4-year period with patients with shorter length of stay at baseline

In the subsample who were hospitalized for less than 2112 days, the model accounted for 30.4% of variance in the change of the mean DUNDRUM-3 over a four-year period (*F* (5, 29) = 3.965, *p* = 0.007). Cognition contributed 20.9% (*F*-change (1, 30) = 8.803, *p* = 0.006) to the model after controlling for age, gender, mean DUNDRUM-3 and cognition at baseline. Adding psychopathology increased the variance explained by 11.8% (*F-*change (1, 29) = 5.777, *p* = 0.023). Three predictors showed statistical significance in the final model. Baseline mean DUNDRUM-3 had the highest beta (β = 0.668, *p* = 0.002), followed by cognition (β = 0.501, *p* = 0.007), and psychopathology (β = − 0.447, *p* = 0.023).

### Hypotheses 4 and 2: Mediation analysis concerning the relationship between neurocognition, change in DUNDRUM-3 and change in HCR-20-C

We used mediation analysis (Fig. 2) between neurocognition (MCCB) as cause, change in violence proneness (HCR-20-C) as outcome and change in mean DUNDRUM-3 as mediator while controlling for baseline HCR-20-C and baseline mean DUNDRUM-3 for the total sample, and applied the same model to the subsamples with length of stay more than five years, and less than five years (Table 5).

**Table 5:** Hayes Process Mediation Model 4 for 10,000 bootstrapped samples: Regression and mediation coefficients

	Change in Y		C1: direct effect of X on Y before mediation		C2: direct effect of X on Y after mediation		A: indirect effect of X on Y mediated via M		B: direct effect of M on Y adjusted for X	
	*R* ^2^	*p*	Unstandardized effect size	95% CI	Unstandardized effect size	95% CI	Unstandardized effect size	95% CI	Unstandardized effect size	95% CI
Total sample n = 69										
Model A										
X = MCCB	0.6241	0.0000	−0.0065	−0.0345, 0.0215	0.0307	− 0.0057, 0.0671	**0.0372**	**0.0129, 0.0663**	**1.8406**	**1.3673, 2.3138**
M = change D-3 mean										
Model B										
X = MCCB	0.6044	0.0000	**0.0121**	**0.0019, 0.0223**	**0.0202**	**0.0064, 0.0340**	0.0081	−0.0020, 0.0168	**0.2637**	**0.1959, 0.3316**
M = change HCR-C										
Less than 5 years *n* = 35										
Model A										
X = MCCB	0.6748	0.0000	−0.0338	−0.0758, 0.0081	0.0213	−0.0312, 0.0738	**0.0551**	**0.0239, 0.0911**	**1.8597**	**1.1937, 2.5257**
M = change D-3 mean										
Model B										
X = MCCB	0.6768	0.0000	**0.0237**	**0.0092, 0.0382**	**0.0296**	**0.0093, 0.0500**	0.0059	−0.0075, 0.0199	**0.2797**	**0.1795, 0.3799**
M = change HCR-C										
More than 5 years *n* = 34										
Model A										
X = MCCB	0.6503	0.0000	0.0240	−0.0146, 0.0626	0.0419	−0.0144, 0.0981	0.0179	−0.0211, 0.0744	**2.099**	**1.3878, 2.8104**
M = change D-3 mean										
Model B										
X = MCCB	0.6315	0.0000	−0.0026	−0.0167, 0.0015	0.0085	−0.0115, 0.0285	0.0111	−0.0018, 0.0260	**0.2653**	**0.1754, 0.3551**
M = change HCR-C										

Note: *CI* Confidence interval, *MCCB* MATRICS consensus cognitive battery, *D-3* DUNDRUM-3 Programme Completion Scale mean total score, *HCR-C* History-Clinical-Risk Management-20 clinical items. In Model A the outcome variable (Y) was change in HCR-20 clinical items during the study. In Model B the outcome variable (Y) was change in DUNDRUM-3 Programme Completion Scale mean total score during the study. X, independent variable; M, mediator variable. All models were controlled for HCR-20 clinical baseline scores and DUNDRUM-3 mean total score at baseline

Model A: Y = HCR-20 clinical score change over four years. Model B: Y = DUNDRUM-3 mean total score change over four years; Results in bold are statistically significant

For the total sample and the shorter stay subsample the effect of MCCB on change in HCR-20-C was completely mediated via change in mean DUNDRUM-3. To explore the direction of the effect, we next tested the MCCB as cause, change in mean DUNDRUM-3 as outcome and mediation between MCCB and DUNDRUM-3 via change in HCR-20-C. There was a direct effect of MCCB on change in mean DUNDRUM-3 and this was not mediated via change in HCR-20-C. MCCB did not affect change in DUNDRUM-3 when controlling for change in HCR-20-C. Therefore this was specific to change in mean DUNDRUM-3 as mediator.

Change in mean DUNDRUM-3 had a significant effect on violence proneness (HCR-20 clinical items) when controlling for cognition for the total sample and subsamples (Table 4). This shows that for the total sample in the mediation model a change of 1 point on the mean DUNDRUM-3 score (range 0 to 4) is related to a change of 1.8 on the HCR-20-C score (range 0 to 10). For the longer stay sub-sample, the relationship between neurocognition and violence proneness was not mediated by change in mean DUNDRUM-3.

## Discussion

Although there is evidence that FMHS reduce the risk of reoffending for patients with schizophrenia and a history of violence it is unclear whether this is attributable to psychosocial treatments [3]. Using the mean DUNDRUM-3 programme completion score, a measure of response to multi-modal treatment, we have shown that patients with schizophrenia or schizoaffective disorder treated within a forensic hospital can achieve both reliable (31%) and reliable and clinically meaningful change (23%). This change concerns their participation, engagement and sustained progress across seven domains of treatment over four years. Changes in the mean DUNDRUM-3 were also associated with changes in a measure of violence proneness, the HCR-20-C. The latter is important because of the link between perceived risk of violence, treatment completion, recovery and longer lengths of stay [22, 23, 28, 30, 31, 45]. It has also been shown that factors related to seriousness of violence and need for higher levels of therapeutic security predict length of stay [46] and the DUNDRUM-3 is calibrated in units relevant to diminishing need for secure care.

Patients hospitalized for less than five years at baseline benefited more from treatment than patients who were hospitalized for more than five years. Cognitive impairment accounted for a significant amount of the variance in patients’ ability to benefit from treatment programs. For the full sample we found no significant change in the HCR-20-D other than a small but significant reduction in the HCR-20-C scale (10.1% achieved reliable change). For the mean DUNDRUM-3 there was a significant mean change though this was also small. However, substantial numbers of forensic patients achieved reliable (31%) and clinically meaningful change (23%) in the mean DUNDRUM-3 score. This was hidden within what appear to be small mean changes. In this study effect sizes for mean change (d) [74, 75] were uninformative, appearing moderate even when actual mean change was small. In contrast, percentages achieving reliable and clinically meaningful change [76] were more informative.

Because many patients experience cognitive impairment and remain symptomatic even when adhering to pharmacotherapy we also investigated the impact of cognition and symptoms on treatment outcome [44, 57]. Cognition accounted for 12.6% of the variance in mean DUNDRUM-3 change and when combined with symptoms the total model accounted for 19.2%.

We hypothesized that those with a longer length of stay at baseline would have less response to treatment than those who were earlier in their hospital stay. We divided the sample using a median split for length of stay approximating to five years. Those who had shorter lengths of stay at the point of entry had a greater change in the DUNDRUM-3. Within the group with shorter length of stay, both cognition and symptoms accounted for larger significant changes on the DUNDRUM-3 scale with cognition accounting for 20.9% of the variance.

Finally, because there was a small but significant change in the HCR-20-C we carried out a mediation analysis to investigate whether cognition affected patients’ violence risk when mediated by treatment change. We compared two models to find a preferred model. The effect of cognition on violence risk was completely mediated by change in mean DUNDRUM-3 within the total sample and the shorter stay subsample. We then found that there was no evidence that change in violence risk mediated ability to benefit from programmes. Violence risk as measured by the HCR-20-C scale was reduced by participation in psychosocial treatments, where participation was in part determined by cognitive ability. Because cognitive problems amongst patients with schizophrenia are thought to occur prior to the onset of psychosis and cannot be ameliorated by medication [39, 40] we have not included symptoms in these mediation models. Our comparison of models suggests a causal pathway from cognition, to ability to make progress in psychosocial treatment as measured by change in the mean DUNDRUM-3, to change in violence risk as measured by the HCR-20-C. For every change of one point mean DUNDRUM-3 score (out of a possible 0 to 4) the HCR-20-C scale changed by 1.8 points (out of a possible 0 to 10).

The findings of this study have theoretical and practical implications. Careful consideration needs to be given to how change is measured. Although the HCR-20 is used to guide treatment and there is some evidence of sensitivity to change between admission and discharge, though not in relation to risk factors such as stress or lack of personal support [27], this study casts doubt on the sensitivity of the HCR-20 to detect change, in line with recent work [28–31]. Whilst the HCR-20 measures risk factors for violence, the mean DUNDRUM-3 focuses on participation in multi-modal clinical interventions and appears to be more sensitive to change. The findings of this study support a model in which multi-modal program completion mediates the relationship between cognition and violence risk. We have shown that change in a measure of multi-modal treatments was related to a reduction in a measure of violence proneness.

Clinicians need to be aware of the impact that cognition and symptoms have on patients’ ability to participate in treatment. Patients may benefit from cognitive remediation therapy [57] and medication review [14] prior to commencing psychosocial interventions.

It remains unclear for how long patients should be hospitalized. Increased duration of treatment appears to benefit outcomes only to a point. Patients require a period of time before making reliable and clinically meaningful changes but within this study, those hospitalized for longer than five years had little further change. The reason for this is unclear. The longer stay group did not have lower scores on neurocognition and had a lower symptom score. Patients hospitalized for longer periods may have complex needs and require different interventions.

### Limitations and strengths

This is a mixed cross-sectional and prospective cohort study. While a fully prospective study of incident cases would have many advantages, it would take many more years to complete. Cross-sectional studies also have advantages in generating timely information [37]. We are gathering information for an eventual incident prospective cohort study. The majority of patients were male. The effect of gender might be underestimated [26, 27]. The findings of this study apply to forensic patients with schizophrenia and schizoaffective disorder at a medium and high level of therapeutic security. The findings may not apply to patients with personality disorders as sole diagnosis. We did not prospectively investigate the number of hours of participation in each program or reduction of violence proneness from the point of admission into the hospital but rather followed up a cross-sectional sample with varying initial lengths of stay. The methodology employed may obscure important temporal and cohort effects on patients’ ability to benefit from treatment. A prospective study of incident cases would need to be conducted over an extensive time period. Only randomized positive controlled trials would help determine the causal relationships between psychosocial interventions and outcomes.

Strengths of this study were that it included most of a national cohort of forensic patients, it used a longitudinal design over four years and employed a range of independently made measures.

We have not relied exclusively on manualised treatment programmes for reasons set out above [16, 17]. There are examples of problems with over-reliance on manualised programmes. Livingston et al. [78] in a demonstration project found that “Despite succeeding in supporting patients’ participation, the intervention had minimal impacts on internalized stigma, personal recovery, personal empowerment, service engagement, therapeutic milieu, and the recovery orientation of services. Peer support demonstrated positive effects on internalized stigma and personal recovery”. Similarly, for the manualised START NOW for offending behaviour the lead validation study [79] showed that in a retrospective cohort analysis of prisoners, the more sessions attended, the greater the benefit. In a secondary analysis of this non-randomised cohort study [80] the authors noted “unmeasured external forces concurrent to START NOW may have influenced later hospitalization (for example, other programming).” We believe this is particularly important for two reasons. First, those who completed other treatments such as substance misuse programmes may have had better outcomes than those who only did START NOW. In keeping with this, Yoon et al. [81] noted that studies of psychological treatments of prisoners that used waiting list or ‘no treatment’ control groups had higher effect sizes than studies using active treatment ‘treatment as usual’ controls. The DUNDRUM-3 addresses this directly by including a range of relevant treatment domains to measure TAU. Secondly, the ‘dose-response’ design [80] is vulnerable to the criticism that those who are most able will attend the most sessions and have the best outcomes, not because of the benefits of treatment but because of some pre-treatment factor [82] such as the common underlying (confounding) factor of neurocognitive ability. It is for this reason that we have adopted the method of mediation analysis with respect to functional neurocognitive ability.

Finally a number of recent studies have examined psychosocial approaches to treatment in this patient group. Dumont et al. [83] showed that an intervention to increase patient engagement by establishing a peer support program, strengthening a patient advisory committee, and creating a patient-led research team did strengthen patient engagement but “strengthening patient engagement contributes toward improving experiences of care in a forensic hospital, but it may have limited effects on outcomes”. Fazel et al. [84] and Chang et al. [85] demonstrated that antipsychotic medication, stimulant medication and medication for addictions reduced violent recidivism in appropriate diagnostic groups; in secondary analyses they showed that completion of psychological treatments targeting general criminal attitudes and substance misuse was associated with reductions in violent reoffending. Further, the associations with these psychological programs were not stronger than those for medications. Specific violence prevention therapies were not associated (positively or negatively) with violent recidivism. Young et al. [86] offer the nuanced interpretation that a stable mental state following antipsychotic medication is a key factor that predicts treatment completion, while the best predictor of treatment effectiveness was attitude towards violence.

Many further questions arise: for example, in a substance misuse programme designed for delivery in 28 group sessions of two hours each, is it better to have one session a week for six months, two sessions a week for three months or three sessions a week for nine weeks? For patients with schizophrenia and cognitive impairment, will such programmes work better after a course of cognitive remediation, meta-cognitive therapy or other elements of an in-patient multi-modal therapy programme in a secure forensic hospital? How many hours per week can patients with schizophrenia and neurocognitive impairments benefit from before becoming cognitively overloaded? These important and unanswered questions could be addressed based on the methodology described here. We suggest that a system capable of assessing change due to a range of multi-modal treatments over sustained periods is a way forward, enabling dismantling studies to determine the benefits of individual elements and enabling randomised positive controlled trials to compare the addition of new treatments to treatment as usual.

Future research should continue to investigate the roles that the method of measurement, cognition, psychopathology, and length of stay have as moderators or mediators of treatment outcome. The ‘dose’ and intensity of treatment and the sequencing of treatments such as cognitive remediation, metacognitive therapies, cognitive behavioural therapy and specific programmes for specific problem behaviours all require further ‘dismantling’ research [16]. It would be advantageous to develop an international consensus on ‘treatment as usual’ within forensic services as a means of benchmarking progress and clinical innovation. Randomized controlled trials can only be meaningfully interpreted when there is a consensus about ‘treatment as usual’. This method of measurement of change in units of reliable and clinically meaningful outcome represents one possible method for comparing outcomes across services, time periods and case mixes, when case mix is controlled for.

## Conclusion

This study set out to employ several methodological advances concerning the conduct of clinical research and service evaluation. These results demonstrated that the methods used capture measures of ‘treatment as usual’ which will form the basis for randomised controlled trials. These findings illustrate the importance of using reliable and clinically meaningful change [30, 32, 76], and more fundamentally, we have shown the value of using measurement scales calibrated in clinically meaningful units [18].

These methods were used to examine the extent to which prolonged treatment for schizophrenia in a secure forensic hospital is beneficial, and the limits of that benefit over time. This study provides a means of assessing the multi-modal, biopsychosocial treatment offered for patients with schizophrenia as ‘treatment as usual’ in a secure forensic hospital. In keeping with those findings which suggest a lower risk of reoffending [3], forensic patients with schizophrenia and schizoaffective disorder appear to benefit from multi-modal psychosocial treatment. A lowered risk of violence is itself an indication of functional recovery in an important domain [22, 23, 44, 45]. This study suggests that the lowered risk of re-offending may be partially attributable to participation and engagement in psychosocial interventions while cognitive impairment and symptom severity reduce the benefits of treatment on violence proneness. The magnitude of this benefit appears to be a function of the phase of treatment [87, 88], the outcome measure used, the use of meaningful units of change, the degree of cognitive impairment, psychopathology, and length of stay. Careful consideration needs to be given to these variables when designing and delivering programs, estimating required duration of treatment, evaluating progress and designing future randomized controlled trials. We believe this is in keeping with the growing movement towards routine outcome measurement and feed-back informed treatment [89].

For the future, the effect of the ‘dose’ of treatment should be considered as duration and frequency of treatment sessions, the sequencing of various modalities (cognitive remediation followed by metacognitive therapy followed by problem focused approaches) and sequencing or concurrence of treatment programmes (physical health, mental health, substance misuse, specific problem behaviours and negative attitudes, self-care and activities of daily living, education occupation and creativity, family and intimate relationships). It would also be useful to examine the ways in which these treatment programme variables interact with patient characteristics such as phase or stage of illness, neurocognitive and social cognitive impairments and symptom severity.

## Abbreviations

A: Indirect effect of X on Y mediated via M
B: Direct effect of M on Y, adjusted for X
C1: Direct effect of X on Y, before mediation via M
C2: Direct effect of X on Y after mediation via M
CI: Confidence interval
CMH: Central Mental Hospital, Dundrum, Dublin
CPZeq: Chlorpromazine equivalents
D: Cohen’s d, effect size
D-3`: DUNDRUM-3 programme completion scale
DSM-IV: Diagnostic and Statistical Manual 4th edition
DUNDRUM toolkit: Dangerousness, Understanding, Recovery and Urgency Management Manual
DUNDRUM-1: Dundrum-1 triage security scale (nine item)
DUNDRUM-3: Dundrum-3 programme completion scale
FMHS: Forensic mental health services
HCR-20: Historical-Clinical-Risk Management Scale-20 Version 2
HCR-20-C: HCR-20 current or clinical items scale
HCR-20-D: HCR-20 dynamic (sum of C and R) items scale
HCR-20-R: HCR-20 risk or future items scale
M: Median
MCCB: MATRICS consensus cognitive battery
MDT: Multi-disciplinary team
NFMHS: National Forensic Mental Health Service
PANSS: Positive and negative symptom scale
RCI: Reliable change index
RMC: Reliable and clinically meaningful change
SCID: Structured clinical interview for DSM disorders
SD: Standard deviation
SPSS-24: Statistical package for the social sciences version 24
x: Mean
